# One Hundred Consecutive Cases of Skin Disease

**Published:** 1891-11

**Authors:** M. B. Hutchins

**Affiliations:** Lecturer on Diseases of the Skin, Atlanta Medical College, Atlanta, Ga.


### ONE HUNDRED CONSECUTIVE CASES OF SKIN DISEASE.

### X.

By M. B. HUTCHINS, M. D.,
Lecturer on Diseases of the Skin, Atlanta Medical College,
Atlanta, Ga.

### ALOPECIA AREATA.

Of this disease there was one case, that of a young physician, age twenty-nine. Hair began coming out, in irregular patches, about a month previous to consultation. Hairs still present in the patches loose, and here and there the hairs had that broken, peg shape something like an exclamation point under the microscope, so characteristic of the hairs usually seen at the edge of a patch in this disease. The disease appeared worse after two weeks use of,

℞. Hydrag. bichlor., gr. xvi,
Sp. vin. rect.,
Aquæ, aa ℥ii,

M.

applied to the scalp with friction. As many of these cases recover spontaneously, I asked the doctor to leave off all treatment, and see what the result would be. He did so. In exactly three weeks he reported full regrowth of the hair.

PRURITUS ANI.

One case of this trouble ; robust young man of twenty-nine. Present more or less for ten years. Never had hemorrhoids, and anus appeared normal upon inspection, save a half pea-sized papular growth of pale color. This was immediately cut off with scissors. Patient believed the troublesome itching to be due to irritation of the stiff hairs around the anus and to the secretion—moisture. Was given,

℞. Saponis viridis, ℥ii,
Sp. vin. rect., ℥i,

M.

to use in washing the part. After washing, and three times a day was to use—

℞. Menthol., gr. x.

Picis liq., ʒii.

Ether. sulph.,

Alcohol, aa ℥i.

M.

I forgot to state that the general health was good and the bowels acted regularly.

The applications relieved the itching, but, in about eight days, produced some irritation. This was subdued by the use of,

℞. Zinc. ox. pulv., ʒij.

Bismuth subnit.,

Amyli., aa ℥i.

M. Sig.—Apply on cotton during the day. To continue application of prescription number two in case the itching tended to recur.

At the end of another week the patient became constipated and there was slight hemorrhoidal congestion. The further history of this case is not known.

The case of a woman, aged thirty-one, who had slight varicose veins of the leg, chronic diarrhœa, and a history of general bad health was diagnosed, primarily, *purpura hemorrhagica.* The escape of blood from the vessels of the affected leg was probably due to the bad circulation. (Purpura is really a symptom, and not a disease, and may be due to various causes.) On left leg, lower part, there were numerous "nail head" sized, purplish points due to extravasated blood, and within this affected area a small irregular ulcer, in the center of which could be seen a minute bluish point, suggesting a ruptured venule. General tonic treatment was instituted, with iron, quinine and strychnine. Locally, chiefly for the benefit of the ulcer,

℞. Ichthyol.,

Glycerine, aa ʒii,

Aquæ, ad. ℥vi,

M.

was ordered used often enough to keep the surface wet. Later it was found necessary to protect the ulcer, partially, with corn-starch. Absorption of extravasation took place rapidly. Besides giving a history of numerous diseases—the patient later gave information which suggested the possibility of syphilitic infection at some distant date. She was given small doses of bichloride of mercury in combination with bismuth and nux vomica. This improved the diarrhœal condition. Ungt. hg. 1 with ungt. zn. ox. 2 did not help the ulcer, as it should have, had syphilis been an element in its production. Under,

℞. Iodoformis, ʒii,

Amyli., ʒvi,

M.

the ulcer nearly healed and the cure was completed with the "ichthyol lotion."

EPIDERMIC HYPERTROPHY, ETC.

Patient, man of forty-one. Condition present about ten months. The carmine border of the lower lip was the seat of this peculiar condition. The epidermic layers of the skin here were markedly thickened, the thickened part loosely attached to skin beneath and the margin sharply limited at the mucous membrane within. The color was the peculiar white of water-soaked, new rubber. There was one small scar. (He stated he had had "fever blisters.") A few slightly enlarged (skin) papillæ were visible in the diseased surface.

Sometimes burned the lip with very hot coffee, which he was in the habit of drinking. General health was good, but he had taken a dose of Simmons' liver regulator every night for five years to keep his bowels "open."

℞. Sp. vin. rect., ℥i,
Acid sal., q. s. ad. solutionem saturatum,
M.

was used at first, with partial success in removing the hypertrophy, but finally ceased to have any effect. *Caustic potash* was then cautiously applied and the thus softened epidermis scraped away with the curette. Finally,

℞. Acid. salicyl., gr. x,
M. Collodii, ℥ss, was used, but not regularly. This, however, finally removed the disease. Just six months later there was a slight return of the disease, but this disappeared, under slightly stronger "salicylic-collodion" application, in less than two weeks. When first treated, the patient was ordered to leave off the "liver medicine," and was given the "cascara and nux mixture." He reported that its use produced a cure of his constipation.

DERMATITIS VENENATA.

This was the case of a young lady of twenty. Symptoms were those usually seen as result of poisoning with "poison oak" or "ivy." Face, nose and right ear inflamed, discharging and forming sero-crusts, just as in an acute eczema, or a dermatitis from any local irritant. On face the following:

℞. Acid. sal., gr. xxx.

Zn. ox., ʒii.

Ungt. simp., ℥ii.

M. Sig.—Keep well applied. In and about ear the following powder was used:

℞. Zn. ox., ʒi.

Bismuth. subnit., ʒii.

Amyli., ℥i.

M. This treatment caused quite rapid improvement. The eruption occurred on the shoulders, in a few days, and the following lotion was ordered:

℞. Calamin. prep., ʒi.

Zn. ox. pulv., ʒii.

Aquæ calcis, ℥ii.

Aquæ, ad. ℥iv.

M. Sig.—Apply to affected surfaces. The patient reported later that she was advised to use "nightshade and cream," and that it "cured" her. It is possible that belladonna may have something of an antidotal effect upon the poison.

---

This ends the report of the hundred cases, the notes of which were made a year ago.

In conclusion, I wish to thank the members of the profession, by whom the majority of these cases were referred to me.

1½ *Edgewood Ave.*

